# Knowledge about dietary supplements and trust in advertising them: Development and validation of the questionnaires and preliminary results of the association between the constructs

**DOI:** 10.1371/journal.pone.0218398

**Published:** 2019-06-24

**Authors:** Michał Seweryn Karbownik, Ewelina Paul, Maja Nowicka, Zuzanna Nowicka, Radosław Przemysław Kowalczyk, Edward Kowalczyk, Tadeusz Pietras

**Affiliations:** 1 Department of Pharmacology and Toxicology, Medical University of Lodz, Łódź, Poland; 2 OSOM STUDIO, Łódź, Poland; 3 Department of Clinical Pharmacology, Medical University of Lodz, Łódź, Poland; 4 Department of Biostatistics and Translational Medicine, Medical University of Lodz, Łódź, Poland; 5 Independent scientist, Berlin, Germany; Iranian Institute for Health Sciences Research, ISLAMIC REPUBLIC OF IRAN

## Abstract

**Background:**

Despite offering little overall benefit and emerging concerns about their safety, dietary supplements have become increasingly popular. Trust in advertising them may contribute to high confidence in dietary supplements in public opinion.

**Aim:**

To develop and validate a screening questionnaire intended for the general public regarding knowledge about dietary supplements and a questionnaire on trust in advertising dietary supplements, and to identify the association between these constructs.

**Materials and methods:**

The development and validation of the measures was overseen by the panels of experts. The conceptual frameworks of the constructs were scientifically well grounded. A set of semi-structured interviews and anonymous web-based surveys was performed. The final questionnaire was applied to 220 non-medically educated people and 121 medically educated people.

**Results:**

A 17-item questionnaire on knowledge about dietary supplements and eight-item questionnaire on trust in advertising dietary supplements were developed. The measures presented satisfactory proof of validity, however, the psychometric properties of the questionnaire on knowledge were modest. Both the knowledge about dietary supplements in the study group and trust in advertising them were low. A significant negative relationship was found between knowledge about dietary supplements and trust in advertising them among the general public (Pearson’s r = -0.42, 95%CI: -0.52 to -0.30, *p*<0.0001). This association was especially pronounced in people who reported not taking dietary supplements (Pearson’s r = -0.61, 95%CI: -0.76 to -0.39, *p*<0.0001).

**Conclusions:**

The extensive advertising of dietary supplements appears to be in conflict with promoting evidence-based knowledge about them, which raises substantial concerns for the public health. The results of the study are only preliminary and require further confirmation and exploration.

## Introduction

Dietary supplements (DS) have become increasingly popular following *inter alia* the seminal work on “vitamins” by Kazimierz Funk [1] and the advocacy of Linus Pauling [2], a double Nobel Prize laureate. DS occupy a well-established and scientifically proven position in correcting malnutrition [3]. However, in high-income countries, DS are also perceived as offering hope for generally healthy people for warding off multiple civilization diseases, extending life expectancy, improving life quality, and even allowing human enhancement [4, 5]. Unfortunately, many of these claims have not been supported by the findings of large-scale high-quality studies, the majority of which found DS to have negligible effects [2, 6, 7]. Vitamin C was found not to protect from common cold in the general community [8], omega-3 fatty acids from cardiovascular events [6, 9], nor antioxidants from cancer [10, 11]. The latter may even have harmful effects [11, 12].

Despite such disappointing data, high confidence in dietary supplements persists among the general public [5, 13]. This persistence may be due to the prevalence of aggressive advertising [14–16] or, more specifically, the extent to which people trust advertising [17, 18]. Consequently, extensive advertising may lead to an accumulation of misleading information [19]. Yet, the economic and social development of a knowledge-based society demands the exchange of correct facts and well-established knowledge [20]. In this light, testing the hypothesis about the link between knowledge about DS and trust in their advertising appears to be of paramount importance. It may help to shape more prudent advertising strategies for DS, make people better informed about them, and contribute to improving health.

The aim of the current study was to report the development and validation of two psychometric tools to measure the above-mentioned constructs: knowledge about dietary supplements and trust in advertising them. The tools will be used in further research to examine the hypothesis about the association between these constructs in detail. Moreover, the aim of the present study is to report preliminary data regarding such association.

## Materials and methods

In the present study, “dietary supplements” were defined as “foodstuffs the purpose of which is to supplement the normal diet and which are concentrated sources of nutrients or other substances with a nutritional or physiological effect, alone or in combination, marketed in dose form”. The definition is consistent with Directive 2002/46/EC of the European Parliament and of the Council on the approximation of the laws of the Member States relating to food supplements, which also applies in Poland [21].

The study was carried out in agreement with the latest version of Declaration of Helsinki. It was approved by the Bioethics Committee of the Medical University of Lodz (number RNN/253/18/KE received on 10 July 2018). All the questionnaires were gathered from mid-July to December 2018 with written or electronic informed consent received each time.

The process of questionnaires development was led by the guidelines reported [22, 23], and by papers describing the development of similar questionnaires [17, 24, 25].

### Development and validation of the questionnaire on knowledge about dietary supplements

**Fig 1:**
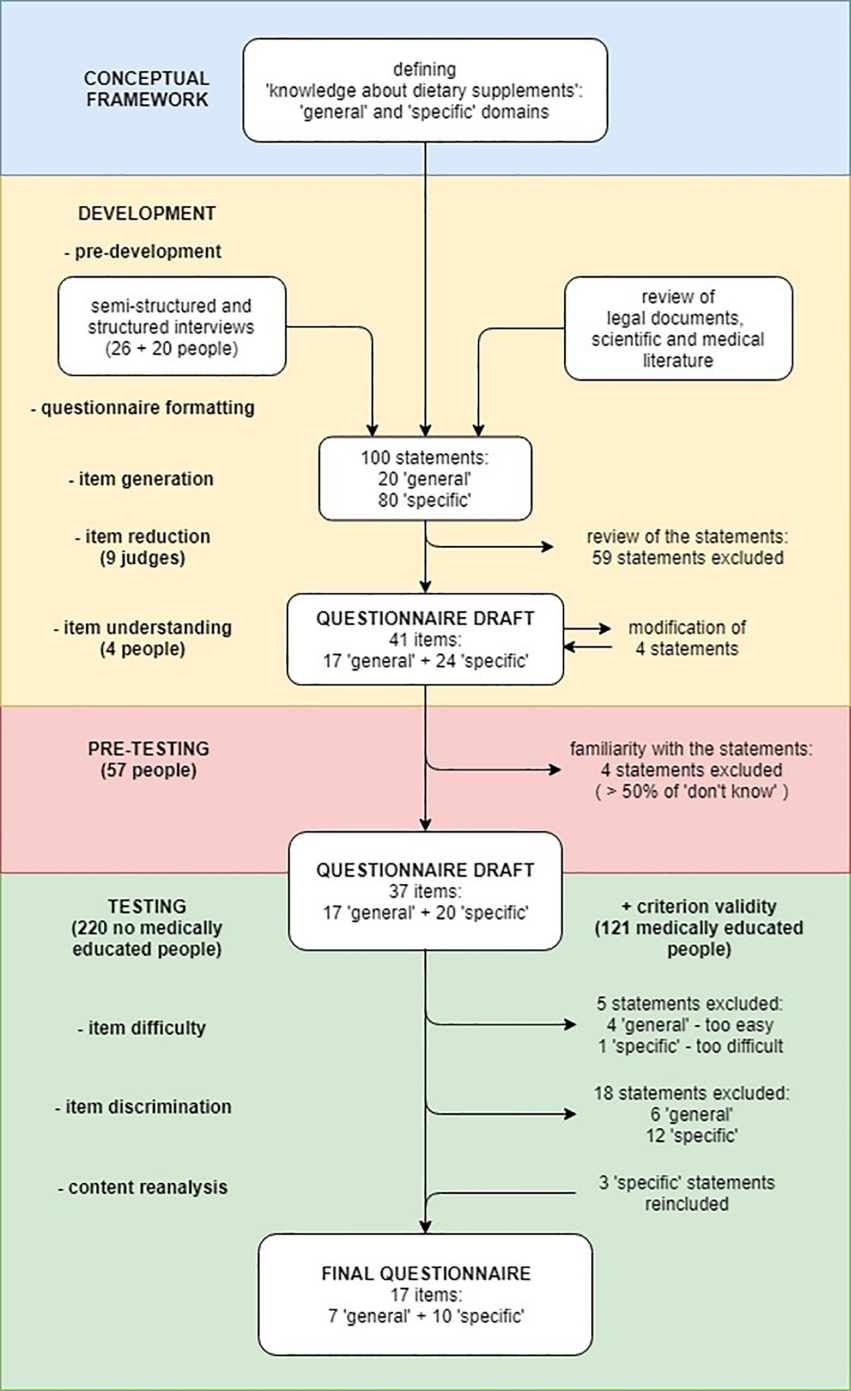
Flowchart illustrating the development of the questionnaire to assess knowledge about dietary supplements (KaDS).

The development of the questionnaire on knowledge is illustrated in the flowchart given in Fig 1. The questionnaire was prepared in four stages: setting the conceptual framework, development, pre-testing and testing. More details on the methods used in its development are included in S1 Supporting Information.

#### Conceptual framework

In order to assure the validity and reliability of testing “knowledge about dietary supplements", abbreviated to KaDS, a tool was developed and validated by a panel of experts. This panel included a pharmacist with academic experience, a pharmacist with clinical experience and a medical doctor with the title of Full Professor. The panel first developed a conceptual framework for the KaDS distinguishing its “general” and “specific” domains. The “general” KaDS was defined as “familiarity with the useful facts concerning legal status of dietary supplements in general”, and the following concepts of this domain were pointed out: 1) definition and 2) registration requirements. On the other hand, the “specific” KaDS was defined as “familiarity with common, scientifically proven and useful facts about popular dietary supplements”, and the following concepts of this domain were highlighted: 1) efficacy (with special attention to this category), 2) safety, 3) pharmacokinetics, and 4) interactions with medicines. The final measure was expected to be a concise questionnaire suitable for screening purposes rather than a detailed evaluation and decision-making tool to examine individual respondents; therefore, the final KaDS questionnaire was restricted to a maximum of 10–20 items.

#### Development

The development of the KaDS questionnaire consisted of several steps:

- Pre-development. A series of pre-development semi-structured and structured interviews was performed with pharmacists and people with no medical education to establish the most commonly-purchased classes of DS, the ingredients which are the most identifiable by the public, and to collect common beliefs about DS.
- Questionnaire formatting. It was decided that each question stem will be presented as a declarative sentence, and that the response format will be of a binary nature (true/false).
- Item generation. Based on the results of the pre-developmental interviews, and a rigorous review of legal documents and scientific and medical literature, 100 statements were drawn up: 20 “general” and 80 “specific”. The statements were reviewed by a specialist in Polish linguistics and further modified according to her suggestions.
- Item reduction. A group of nine independent competent judges were asked to evaluate each statement in terms of its relevance and its value to public knowledge. Based on their opinion, 59 less relevant statements were deleted [22], and the remaining 41 were passed to the next step of the development process.
- Item understanding. The retained 41 statements were tested with regard to their degree of understanding by the public [26]. The vast majority of the statements were understood as assumed by the panel of experts, but four required further clarification and were modified accordingly.

#### Pre-testing

The aims of the pre-testing stage were to identify the statements which are less familiar to the general public and to obtain data on face validity. The 41-item draft of the KaDS questionnaire was prepared in a web-based form (Google Forms, Google, Mountain View, CA, USA) and administered to a sample of 57 people with no medical education. An additional “don’t know” option was added to the true/false response format.

Four statements received more than 50% “don’t know” answers; these were hence assessed as unfamiliar to the public, and rejected from the questionnaire. The remaining 37 statements were retained. Feedback provided by the respondents indicated that the KaDS questionnaire was clear and comprehensive.

#### Testing

The testing stage assessed the psychometric characteristics of the items and pruned those with less psychometric relevance. The 37-item draft of the KaDS questionnaire, obtained in the pre-testing stage, was again prepared in a web-based form (Google Form) with true/false response format, but without the “don’t know” option. This draft was combined with the recently-developed eight-item measure of “trust in advertising dietary supplements” (see below) to assess the association between the KaDS and trust in advertising DS. The survey was administered to a sample of 220 people with no medical education. Both the original and English language versions of the survey are available in S1 Survey.

Keeping the conceptual framework of the scale in mind, the questionnaire was refined with regard to the following psychometric properties:

- item difficulty–excessively difficult (> 90% of wrong answers) and easy (< 10% of wrong answers) statements were excluded [23, 27, 28],
- item discrimination–statements with an item discrimination (measured as a corrected item-total correlation with assumed subscales) of less than 0.20 were excluded [23],
- maximization of internal consistency [29],
- balancing the number of “true” and “false” correct answers [30] and content reanalysis.

The final version of the KaDS questionnaire consisted of 17 items. Exploratory and confirmatory factor analysis was performed to assess the consistency of the final questionnaire structure. To ensure the criterion validity of the final KaDS questionnaire, it was also applied to 121 medically educated people (healthcare practitioners and medical students). Criterion validity was assessed by comparing the mean test score between medically and non-medically educated people. Additionally, the correlation between the test score and a year of study was checked among a group of medical students.

The data of 220 responses of non-medically educated people and 121 responses of medically educated people for the testing of the KaDS questionnaire was deposited in Mendeley Data repository (http://dx.doi.org/10.17632/h8f4t3ktd5.1).

### Development and validation of questionnaire on trust in advertising dietary supplements

**Fig 2:**
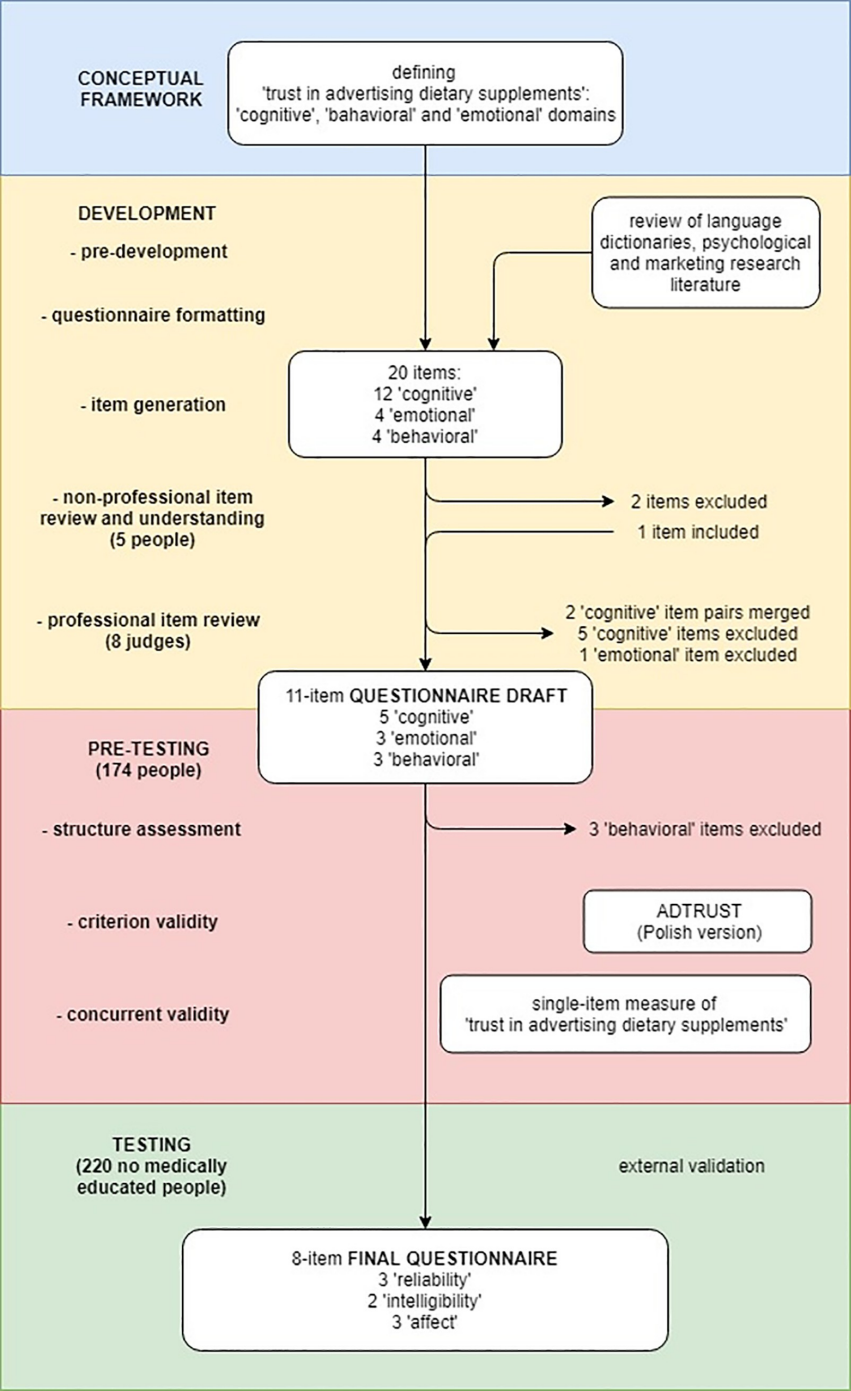
Flowchart illustrating the development of the questionnaire to assess trust in advertising dietary supplements (TiADS).

The development of the questionnaire on trust is illustrated in the flowchart given in Fig 2. The questionnaire was prepared in four stages: setting the conceptual framework, development, pre-testing and testing. More details on the methods used in its development are included in S2 Supporting Information.

#### Conceptual framework

A panel of experts was formed to develop a tool for testing “trust in advertising dietary supplements", abbreviated to TiADS, and ensure its validity and reliability. The panel included a psychologist with academic experience, a psychologist with clinical experience, and two marketing specialists. The panel first defined advertising dietary supplements as “any paid form of non-personal presentation and promotion of goods, services, or ideas (*i*.*e*. dietary supplements) by an identified sponsor” [31]. Various sources of advertising were included, *inter alia* television, radio, internet/mobile and newspapers/magazines. The panel then reviewed the popular Polish and English language dictionaries as well as psychological and marketing research literature [17, 32–34] to define the construct of TiADS as “a belief that advertising is a credible source of information about dietary supplements, arouses emotions and willingness to act on the basis of information conveyed by advertisements”. This definition is consistent with prior conceptualizations and identifies the cognitive, behavioral and emotional domains of the construct.

#### Development

The development of the TiADS questionnaire consisted of several steps:

- Pre-development. In-depth lexical analysis of popular Polish and English language dictionaries as well as rigorous review of psychological and marketing research literature was performed to further explore the meaning of TiADS domains [17, 32–34].
- Questionnaire formatting. It was decided that a semantic differential scale will be used. The scale points were numbered from 1 (distrust) to 5 (trust).
- Item generation. In addition to the instructions for using the questionnaire, the panel of experts developed 12 items consisting of opposing adjectives concerning the information conveyed by the DS advertisements (cognitive domain), and eight items consisting of opposing expressions characterizing DS advertisements themselves (four belonging to the behavioral domain and four to the emotional domain). These 20 items were further reviewed by a specialist in Polish linguistics and modified according to her suggestions.
- Non-professional item review and understanding. Five non-specialists were recruited to review the developed items. They were asked to describe in their own words how they understood each item and were interviewed on how much they believed each item was relevant to DS advertising. Moreover, the respondents were asked to develop their own items according to the guidelines provided. The items not understood in the assumed way or assessed as irrelevant/redundant to DS advertising were deleted, whereas the items developed by non-specialists, and which were graded by the panel of experts as at least satisfactorily suited to the definition of the construct were added. This step resulted in retaining 19 items.
- Professional item review. Afterwards, eight independent competent judges were also asked to review the TiADS questionnaire. The judges were acquainted with the conceptual framework of the TiADS construct and asked to evaluate each statement in terms of its relevance to DS advertising. Irrelevant items were deleted. As a result of this review, 11 items were retained in the final draft of TiADS questionnaire.

#### Pre-testing

Questionnaire pre-testing was performed to assess the structure of the TiADS construct and prune the psychometrically less suitable items. The 11-item draft of the TiADS questionnaire was administered in a web-based platform (Google Forms) to 174 respondents. The choice of the items to be retained in the final TiADS questionnaire was based on exploratory factor analysis with subsequent internal consistency assessment [29].

This stage also evaluated the criterion and concurrent validity of the final TiADS measure. Criterion validity was assessed by comparing the results of the TiADS questionnaire with those of the ADTRUST scale. ADTRUST is a 20-item questionnaire developed by Soh *et al*. [17] to measure “trust in advertising” in general. Concurrent validity was assessed by comparing the TiADS scores with the results of a single-item measure of “trust in advertising dietary supplements”. Both the additional measures were included to the web-based survey subjected to 174 respondents.

As a result of item pruning, the final TiADS questionnaire consisted of eight items. The data of for this stage of the TiADS questionnaire analysis was deposited in Mendeley Data repository (http://dx.doi.org/10.17632/nfw68tgw82.1).

#### Testing

During this stage, the final eight-item TiADS questionnaire was externally validated in a separate sample of respondents reflecting the target population. Confirmatory factor analysis was used to verify the established before structure of the final TiADS questionnaire. The TiADS questionnaire was combined with the KaDS test (see above) for preparation for online distribution (Google Forms). This survey was applied to 220 people with no medical education as well as to 121 medically-educated people (see above). Both the original and English language versions of the survey are available in S1 Survey.

The data for testing the TiADS questionnaire was deposited in Mendeley Data repository (http://dx.doi.org/10.17632/h8f4t3ktd5.1).

### Other tested variables

The questions on the following sociodemographic variables were included to the web-based tests throughout the study: sex (operationalized as a categorical variable: 0 = male, 1 = female), age (operationalized as a continuous variable), education level (operationalized as an ordinal variable: 1 = primary, 2 = secondary, 3 = higher-bachelor, 4 = higher-master, 5 = higher-doctorate) and perceived socioeconomic status (operationalized as an ordinal variable: 1 = low, 2 = middle, 3 = high). Respondents were also asked “whether they take DS” (the variable operationalized as an ordinal: 0 = not taking, 1 = taking from time to time, 2 = taking every day) and “whether they had contact with DS advertisements within the past week” (the variable operationalized as an ordinal: 0 = no, 1 = yes).

### Data analysis

Descriptive statistics involved mean ± standard deviation or mean and 95% confidence intervals (95%CI), if not stated otherwise. Although the Likert and semantic differential scale data should be perceived as ordinal variables, the analyses in this study were performed with parametric tests such as Pearson’s *r* (further referred to as “r”), Student’s *t*-test and other general linear model (GLM) analyses. This was to allow for multivariate modeling. Each time, if possible, however, corresponding non-parametric tests were performed and they all yielded very similar results to the parametric ones. Adjustment for potential confounders was performed by including all the covariates to the GLM, linearly linked to dependent variable. Testing multiple hypotheses was not a subject for significance level correction because of the explorative and preliminary nature of the research. The proportion of missing data in the analyzed databases were acceptably low: 0.60% missing data in the database of 57 responses (KaDS pre-testing), 0.42% missing data in the database of 220 responses (KaDS and TiADS testing in non-medically educated people), 0.47% missing data in the database of 121 responses (KaDS and TiADS testing in medically educated people), 0.73% missing data in the database of 174 responses (TiADS pre-testing). Because of low proportions of missing values, all the analyses were based on complete cases only. *P*-values were presented with two significant figures and rounded to maximum four decimal places. *P*-values below 0.05 were considered statistically significant. The analysis was performed using STATISTICA 13.1 Software (StatSoft, Tulsa, OK, USA).

## Results

The sociodemographic characteristics of the group of 220 people with no medical education who completed the KaDS and TiADS questionnaires in their testing stages is given in Table 1.

**Table 1:** The characteristics of respondents who participated in the testing stage of the development of questionnaires.

Variable	Mean (standard deviation) or n (frequency)
Sex	
Male	60 (27.3%)
Female	160 (72.7%)
Age (n = 215)*	
[years]	36.5 (12.3), range:18–77
Education	
Primary	1 (0.5%)
Secondary	54 (24.5%)
Higher–Bachelor	34 (15.5%)
Higher–Master	129 (58.6%)
Higher–Doctorate	2 (0.9%)
Perceived socioeconomic status	
Low	17 (7.7%)
Middle	166 (75.5%)
High	37 (16.8%)
Do you take dietary supplements? (n = 219)*	
No	52 (23.7%)
Yes, from time to time	123 (56.2%)
Yes, everyday	44 (20.1%)
Did you have contact with DS advertisements within the past week?	
No	101 (45.9%)
Yes	119 (54.1%)

* In case of missing value for a variable, the total number of responses was indicated.

### Questionnaire on knowledge about dietary supplements

#### Psychometric properties of the final questionnaire

The following psychometric results of the final KaDS test are presented in Table 2: the mean grade awarded to each of the final 17 items of the KaDS questionnaire by the competent judges during the item reduction step, familiarity of the general public with the statements assessed in the pre-testing stage, the results of the item analyses performed in the testing stage, and the results of exploratory and confirmatory factor analyses, as well as their internal consistency indices. Psychometric analysis found the structure of the KaDS questionnaire indices to be modest. Consequently, the 17-item test appears not fully adequate for detailed knowledge evaluation and decision-making in individual respondents, but may be successfully used for rapid screening purposes.

**Table 2:** Psychometric properties of the items in the knowledge about dietary supplements (KaDS) questionnaire.

No	English translation of each retained statement	True (T) or False (F)	Mean grade by competent judges^1^ (SD)	Familiarity^2^	Difficulty^3^	Discrimination^4^	Exploratory Factor Analysis^5^		Confirmatory Factor Analysis^6^ Parameter estimate (95%CI)	Reference supporting the statement
							Factor 1 loadings	Factor 2 loadings		
7-item “general” subscale (Kuder-Richardson 20 = 0.77; skewness = -0.17, 95%CI: -0.50 to 0.15; kurtosis = -1.22, 95%CI: -1.87 to -0.58)										
1	Before being marketed, dietary supplements must be tested for efficacy and safety.	F	4.67 (0.71)	49/57 (86%)	100/220 (45.5%)	0.67	**0.81**	0.02	0.40 (0.34 to 0.46) *p*<0.0001	[16, 35–37]
2	An ingredient may be sold both as a medicine or as a dietary supplement.	T	4.56 (0.73)	41/57 (72%)	81/220 (36.8%)	0.20	**0.25**	-0.37	0.08 (0.01 to 0.15) *p* = 0.012	
3	The quality of dietary supplements is routinely tested before being marketed.	F	4.44 (1.01)	42/56 (75%)	93/219 (42.5%)	0.59	**0.76**	0.11	0.36 (0.30 to 0.42) *p*<0.0001	
4	The packaging of dietary supplements must contain information on possible adverse effects resulting from their use.	F	4.22 (1.39)	48/57 (84%)	112/218 (51.4%)	0.52	**0.68**	-0.24	0.28 (0.21 to 0.35) *p*<0.0001	
5	Dietary supplements are food.	T	4.78 (0.44)	41/56 (73%)	115/219 (52.5%)	0.28	**0.39**	-0.14	0.14 (0.07 to 0.21) *p* = 0.0002	
6	Dietary supplement registration requires assessing the composition of the product by the appropriate supervisory body.	F	4.78 (0.44)	46/57 (81%)	114/220 (51.8%)	0.63	**0.76**	-0.05	0.35 (0.29 to 0.41) *p*<0.0001	
7	All dietary supplements sold in pharmacies have been tested for safety.	F	4.67 (0.71)	43/56 (77%)	73/219 (33.3%)	0.58	**0.74**	0.13	0.34 (0.28 to 0.40) *p*<0.0001	
10-item “specific” subscale (Kuder-Richardson 20 = 0.54; skewness = 0.23, 95%CI: -0.09 to 0.56; kurtosis = 0.04, 95%CI: -0.61 to 0.69)										
8	Taking vitamin and mineral supplements prevents diseases in healthy people.	F	4.44 (1.13)	49/57 (86%)	94/220 (42.7%)	0.25	0.24	**0.27**	0.19 (0.10 to 0.27) *p*<0.0001	[2, 6–10, 35, 36]
9	In the elderly, taking vitamin D reduces the risk of bone fractures.	F	4.44 (1.01)	45/56 (80%)	154/219 (70.3%)	0.32	-0.13	**0.65**	0.12 (0.05 to 0.20) *p* = 0.0010	[38, 39]
10	In the elderly, the use of magnesium preparations prevents muscle cramps.	F	4.29 (0.76)	45/57 (79%)	169/219 (77.2%)	0.21	-0.04	**0.60**	0.17 (0.09 to 0.25) *p*<0.0001	[40, 41]
11	Taking dietary supplements containing calcium reduces the risk of bone fractures in the elderly.	F	4.50 (0.53)	45/57 (79%)	150/220 (68.2%)	0.28	0.16	**0.55**	0.22 (0.14 to 0.33) *p*<0.0001	[42, 43]
12	The use of multivitamin preparations protects against heart diseases.	F	4.50 (0.84)	35/57 (61%)	41/218 (18.8%)	0.25	0.22	**0.39**	0.14 (0.07 to 0.21) *p*<0.0001	[44, 45]
13	The use of antioxidants prevents the development of cancer.	F	4.00 (1.32)	35/57 (61%)	134/220 (60.9%)	0.26	0.02	**0.42**	0.14 (0.05 to 0.22) *p* = 0.0020	[10, 11]
14	Regular use of vitamin C reduces the risk of catching a cold.	F	4.50 (0.93)	48/57 (84%)	176/219 (80.4%)	0.32	0.35	**0.47**	0.20 (0.13 to 0.27) *p*<0.0001	[8, 46]
15	Taking excessive amounts of magnesium supplements can cause diarrhea and nausea.^7^	T	4.12 (0.99)	34/57 (60%)	44/220 (20.0%)	0.12	0.01	**0.03**	0.04 (-0.03 to 0.11) *p* = 0.24	[47]
16	Vitamin C naturally present in food is better assimilated than synthetic.^7^	F	4.44 (0.88)	52/57 (91%)	196/220 (89.1%)	0.09	-0.04	**0.10**	0.03 (-0.03 to 0.08) *p* = 0.35	[48]
17	People with kidney disease should not use high doses of vitamin C.^7^	T	4.22 (0.83)	29/57 (51%)	80/220 (36.4%)	0.18	0.05	**0.23**	0.09 (0.00 to 0.17) *p* = 0.044	[49, 50]
Eigenvalue							3.31	2.00		
Variance explained							19.5%	11.8%		

^1^ A grade by competent judges was ranging from “5” (very relevant and important for the public to know) to “1” (very irrelevant and unimportant).

^2^ Familiarity–defined as 1-fraction of “don’t know” answers.

^3^ Difficulty–defined as fraction of wrong answers.

^4^ Discrimination–corrected item-total correlation.

^5^ Varimax rotation was used to enhance the dissimilarity of the extracted factors

^6^ Confirmatory factor analysis: χ^2^(118) = 225.3, *p* < 0.0001, χ^2^/df = 1.91, adjusted goodness of fit index = 0.85, Tucker-Lewis index = 0.78, Bollen’s Incremental Fit Index = 0.81, Bentler’s Comparative Fit Index = 0.81, Root Mean Square Error of Approximation = 0.070 (90%CI: 0.058–0.083), Standardized Root Mean Square Residual = 0.078. The subscales: “general” and “specific” were correlated with each other: CFA parameter estimate for the correlation = 0.29 (95%CI: 0.11–0.48), *p* = 0.0019.

^7^ These items were retained in the final questionnaire despite unfavourable psychometric properties in order to restore content validity.

Internal consistency of the questionnaire estimated by the McDonald’s omega = 0.79.

The finally developed 17-item KaDS questionnaire satisfactorily fulfils the assumed requirements of criterion validity. The mean number of correct answers for the questionnaire in the group of respondents with medical education was higher than that of no medically educated by 2.1 (95%CI: 1.4 to 2.7) points (*p*<0.0001, Student’s *t*-test). The difference remained significant also after adjustment for multiple potential confounders, such as sex, age, education, perceived socioeconomic status and taking DS (2.0, 95%CI: 1.3 to 2.8, *p*<0.0001; GLM with KaDS as a dependent variable, medical education and covariates as independent variables). In the group of medical students (n = 77), the scores in final KaDS questionnaire was also found to positively correlate with a year of study: r = 0.39 (95%CI: 0.19 to 0.57), *p* = 0.0004.

#### Level of knowledge about dietary supplements

Knowledge about dietary supplements in non-medically educated people was low. The mean number of correct answers in the 17-item questionnaire was 8.3 (95%CI: 7.9 to 8.7). The highest proportion of incorrect answers was found for questions related to the efficacy and absorption of the most well-known DS: vitamin C, magnesium, vitamin D and calcium (see Table 2). This reflects the discrepancy between common beliefs and scientific facts.

Among the tested covariates in the group of people with no medical education, the KaDS total score was positively correlated with respondent’s level of education (r = 0.17, *p* = 0.014) and negatively correlated with the use of DS (r = -0.21, *p* = 0.0024). None of the other tested sociodemographic variables were correlated with KaDS total score: sex (*p* = 0.35), age (*p* = 0.37), perceived socioeconomic status (*p* = 0.79).

### Questionnaire on trust in advertising dietary supplements

#### Psychometric properties of the final questionnaire

The results of exploratory factor analysis performed in the pre-testing stage of TiADS questionnaire development (174 respondents) as well as the results of confirmatory factor analysis performed in the subsequent stage of TiADS questionnaire testing (220 respondents with no medical education) are reported in Table 3. Internal consistency measures and distribution of the responses from the testing phase are also presented there. Confirmatory factor analysis found the structure of the final TiADS to be consistent with previous findings. The model fit, non-centrality-based indices and item loadings were adequate; this supports the robustness of the results.

**Table 3:** Psychometric properties of the items in the trust in advertising dietary supplements (TiADS) questionnaire.

English translation of each retained item	Pre-testing stage (n = 174)			Testing stage (n = 220)	
	Factor loadings in exploratory factor analysis			Confirmatory factor analysis^1^ parameter estimate (95%CI)	Median (25th-75th percentile) answer to the item^2^
	“Reliability”	“Intelligibility”	“Affect”		
“Reliability” (Cronbach’s alpha = 0.82; skewness = 0.43, 95%CI: 0.10 to 0.75; kurtosis = -0.14, 95%CI: -0.78 to 0.51)^3^					
“true (reliable)—untrue (unreliable)”	**0.80**	0.02	0.32	0.74 (0.63–0.85) *p*<0.0001	2 (1–3)
“credible—not trustworthy”	**0.88**	0.11	0.21	0.91 (0.79–1.02) *p*<0.0001	2 (1–3)
“full (comprehensive)—incomplete (incomprehensive)”	**0.84**	0.16	0.15	0.72 (0.58–0.86) *p*<0.0001	2 (1–3)
“Intelligibility” (Cronbach’s alpha = 0.59; skewness = 0.15, 95%CI: -0.17 to 0.47; kurtosis = -0.43, 95%CI: -1.07 to 0.21) ^3^					
“understandable—unclear”	0.01	**0.90**	-0.00	0.68 (0.46–0.90) *p*<0.0001	3 (3–4)
“unambiguous—ambiguous”	0.34	**0.73**	0.20	0.80 (0.56–1.04) *p*<0.0001	3 (2–4)
“Affect” (Cronbach’s alpha = 0.88, skewness = 0.92, 95%CI: 0.60 to 1.24; kurtosis = 0.11, 95%CI: -0.53 to 0.75) ^3^					
“I like them—I don’t like them”	0.19	0.07	**0.91**	0.91 (0.78–1.03) *p*<0.0001	1 (1–3)
“I enjoy them—they annoy me”	0.17	0.09	**0.93**	0.94 (0.83–1.05) *p*<0.0001	1 (1–2)
“they should be broadcast more often—their emission should be limited”	0.27	-0.02	**0.78**	0.77 (0.65–0.88) *p*<0.0001	1 (1–2)
Eigenvalue	2.38	1.40	2.51		
Variance explained	29.7%	17.6%	31.3%		

^1^ Confirmatory factor analysis: χ^2^(17) = 30.6, *p* = 0.022, χ^2^/df = 1.80, adjusted goodness of fit index = 0.93, Tucker-Lewis Index = 0.97, Bollen’s Incremental Fit Index = 0.98, Bentler’s Comparative Fit Index = 0.98, Root Mean Square Error of approximation = 0.058 (90%CI: 0.016–0.093), Standardized Root Mean Square Residual = 0.040. The TiADS dimensions were highly correlated with each other. CFA parameter estimates for the correlations: “reliability” and “intelligibility”: 0.48 (95%CI: 0.31–0.64), *p* < 0.0001, “reliability” and “affect”: 0.49 (95%CI: 0.37–0.61), *p* < 0.0001, “intelligibility” and “affect”: 0.30 (95%CI: 0.12–0.48), *p* = 0.0008.

^2^ 5 –strongly agree with the expression in the left, 1 –strongly agree with the expression in the right

^3^ Estimated in the testing stage (n = 220).

Internal consistency of the questionnaire estimated in the testing stage by the McDonald’s omega = 0.92.

The eight-item TiADS questionnaire was assessed for criterion and concurrent validity in the pre-testing stage of questionnaire development. TiADS scores were found to be significantly correlated with the Polish-version ADTRUST measure (r = 0.45, 95%CI: 0.32 to 0.56, *p*<0.0001) and with the answer to a single question on “how much does a respondent trust the information conveyed in dietary supplement advertising” (r = 0.55, 95%CI: 0.45 to 0.66, *p*<0.0001). The above correlations were not significantly affected by the medical education of the respondents (*p*-values for the interactions between the slopes were 0.41 and 0.83, respectively; GLM with TiADS as a dependent variable and the following independent variables: ADTRUST (or single-item measure of trust), medical education–as a three-level categorical variable–and their two-way interaction). This supports criterion and concurrent validity of the final TiADS questionnaire.

#### Level of trust in advertising dietary supplements

The analysis of the results for question on “trust in the information conveyed in dietary supplement advertising” (pre-testing stage of questionnaire development) in non-medically educated people only (n = 77), indicated that such trust was assessed as low, reaching a median of 3 (25^th^-75^th^ percentile: 2–4) in a seven-point scale with anchors of”absolutely trust” (7)—“don’t trust at all” (1), with 4 being neutral. Only four people (5.2%) rated their trust in DS advertising as more favorable than neutral. Similar results were obtained in the testing stage (n = 220), which indicate a particularly low level of trust in its “affective” dimension (see Table 3).

The results of testing stage indicated also that TiADS is significantly associated with taking dietary supplements (r = 0.26, 95%CI: 0.13 to 0.38, *p* = 0.0001), but not with any of the tested sociodemographic variables: sex (*p* = 0.88), education level (*p* = 0.23) or perceived socioeconomic status (*p* = 0.60), indicating a trend only for older people to be more trusting (r = 0.13, 95%CI: -0.00 to 0.26, *p* = 0.055). Applying the TiADS questionnaire additionally to 121 medically educated people revealed that they trusted DS advertising significantly less than those without a medical education (15.9 ± 4.7 points vs 18.8 ± 5.5 points, *p*<0.0001, Student’s *t*-test), and the difference remained significant after adjustment for all above mentioned covariates (16.3 ± 5.8 points *vs*. 18.6 ± 5.3 points, *p* = 0.0004; GLM with TiADS as a dependent variable, medical education and covariates as independent variables). Both groups, *i*.*e*. medically educated and non-medically educated people, displayed similar relationships between TiADS and all tested sociodemographic variables, with age being significantly linked to trust in medically-educated people (r = 0.19, 95%CI: 0.01 to 0.36, *p* = 0.041). TiADS was also significantly associated with taking dietary supplements in medically-educated people (r = 0.23, 95%CI: 0.05 to 0.40, *p* = 0.012).

### Knowledge about dietary supplements and trust in advertising them

#### Association between the constructs

**Fig 3:**
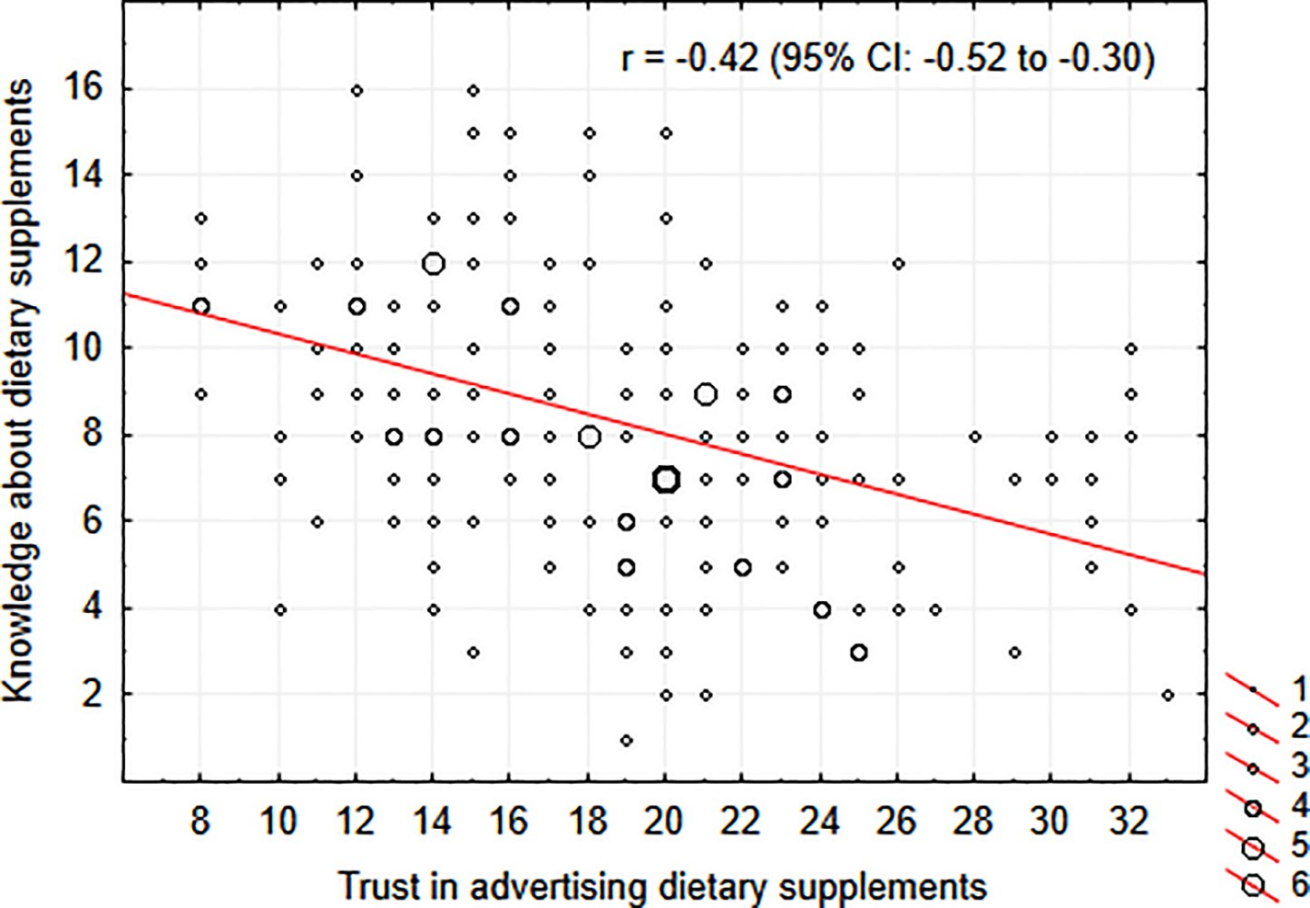
The association between knowledge about dietary supplements and trust in advertising them in non-medially educated people. Knowledge about dietary supplements was measured as a total score in the 17-item questionnaire, whereas trust in advertising dietary supplements with the eight-item questionnaire. The size of the circles represents the number of cases with the given coordinates.

In the group of respondents with no medical education (n = 220), a significant negative association between KaDS and TiADS was detected: r = -0.42 (95%CI: -0.52 to -0.30), *p*<0.0001. The KaDS-TiADS association is illustrated in Fig 3. All the associations between particular KaDS and TiADS subscales were also significantly negative (Table 4). The significance of the association between KaDS and TiADS does not depend much on the choice of items to the final questionnaire, as a significant association was found between the sum of all 32 KaDS items considered for the final version of the questionnaire (testing stage after removal of too easy and too difficult items) and total TiADS score (r = -0.28, 95%CI: -0.40 to -0.15, *p*<0.0001). After splitting the sample of 220 respondents into two subgroups of low and high trust based on median TiADS score, it was found that the 17-item KaDS score of the high trust group was 2.7 (95%CI: 2.0 to 3.5) points lower than that of the low trust group. In KaDS subscale analysis, the differences were: 1.3 (95%CI: 0.7 to 1.9) points for the 7-item “general” KaDS, and 1.4 (95%CI: 1.2 to 1.6) points for the 10-item “specific” KaDS. Hence, the effect of TiADS on KaDS may be at least as high as the effect of formal medical education (see above).

**Table 4:** The association between the subscales of knowledge about dietary supplements and trust in advertising them in the general public. The values presented include Pearson’s correlation coefficient (95% confidence intervals) and *p*-value. *P*-values for each of the comparisons between the correlation coefficients for a given KaDS-TiADS subscale were higher than 0.05.

	“General” KaDS	“Specific” KaDS	KaDS total
TiADS “Reliability”	-0.26 (-0.38 to -0.13) *p* = 0.0001	-0.34 (-0.45 to -0.21) *p*<0.0001	-0.38 (-0.49 to -0.26) *p*<0.0001
TiADS “Intelligibility”	-0.19 (-0.32 to -0.06) *p* = 0.0049	-0.23 (-0.35 to -0.10) *p* = 0.0006	-0.29 (-0.41 to -0.17) *p*<0.0001
TiADS “Affect”	-0.16 (-0.28 to -0.02) *p* = 0.021	-0.23 (-0.35 to -0.10) *p* = 0.0007	-0.25 (-0.37 to -0.12) *p* = 0.0005
TiADS total	-0.25 (-0.38 to -0.12) *p* = 0.0002	-0.39 (-0.50 to -0.27) *p*<0.0001	-0.42 (-0.52 to -0.30) *p*<0.0001

KaDS–knowledge about dietary supplements

TiADS–trust in advertising dietary supplements

Similar negative links between KaDS and TiADS were observed in medically-educated people (n = 121): r = -0.34 (95%CI: -0.49 to -0.17), *p* = 0.0002 for the KaDS total score, r = -0.18 (95%CI: -0.35 to -0.00), *p* = 0.049 for “general” knowledge, and r = -0.33 (95%CI: -0.48 to -0.16), *p* = 0.0003 for “specific” knowledge. *P*-values for the interactions between the corresponding KaDS-TiADS slopes of people with and without medical education were 0.68, 0.48, and 0.89 for KaDS total score, “general” KaDS, and “specific” KaDS, respectively. This similarity of the associations supports the robustness of the results.

#### Variables affecting the extent of KaDS-TiADS association

Interestingly, the use of DS was found to influence the association of KaDS-TiADS in non-medically educated people. This association was significantly more negative in people who reported not taking DS (r = -0.61, 95%CI: -0.76 to -0.39, *p*<0.0001) than those taking DS occasionally (r = -0.30, *p* = 0.0011) or everyday (r = -0.37, *p* = 0.018). The *p*-value for the interaction between the slopes was 0.020, and remained low (0.0062) after adjustment for the covariates: sex, age, education and socioeconomic status (GLM with KaDS as a dependent variable and the following independent variables: TiADS, taking DS (operationalized as a continuous variable), their two-way interaction, and, optionally, all the covariates). No other tested variables were found to significantly affect the extent of the association between KaDS and TiADS (*p*-values for the interaction between the slopes were 0.14 for sex, 0.17 for education, 0.40 for age, 0.91 for socioeconomic status; GLM with KaDS as a dependent variable, and the following independent variables: TiADS, a sociodemografic variable and their two-way interaction).

#### Having recent contact with dietary supplement advertisements and knowledge about dietary supplements

Among those without a medical education, having recent contact (within the past week) with DS advertisements was hypothesized to strengthen the potential link between TiADS and KaDS or to demonstrate its own effect on KaDS. The negative correlation between the trust and knowledge seemed to be more profound in the respondents who declared having recent contact with DS advertisements than those who did not (Table 5); however, the interaction between having recent contact with DS advertisements and TiADS was found to be significant only for “specific” domain of KaDS, which is the main content of DS advertisements [36].

**Table 5:** The association between the knowledge about dietary supplements and trust in advertising them among the general public: Those in recent contact with advertisements and those who are not. The values presented include Pearson’s correlation coefficient (95% confidence intervals) and *p*-value.

Having recent contact with DS advertisements	Association between TiADS and		
	“general” KaDS	“specific” KaDS	KaDS total
Yes	-0.29 (-0.45 to -0.11) *p* = 0.0023	-0.50 (-0.63 to -0.35) *p*<0.0001	-0.50 (-0.63 to -0.34) *p*<0.0001
No	-0.22 (-0.40 to -0.02) *p* = 0.030	-0.23 (-0.41 to -0.03) *p* = 0.025	-0.30 (-0.47 to -0.10) *p* = 0.0035
Interaction between the slopes*	*p* = 0.88	*p* = 0.028	*p* = 0.15

TiADS–trust in advertising dietary supplements

KaDS–knowledge about dietary supplements

* general linear model with KaDS as a dependent variable, and the following independent variables: TiADS, having recent contact and their two-way interaction

Similarly, the analysis of covariance (a model with different slopes for “specific” KaDS only) indicated that having recent contact with DS advertisements had a significant negative effect on the “specific” KaDS score (*p* = 0.039); however, the effect size was extremely low (-0.04 point in the 10-item “specific” subscale). The effect of recent contact with DS advertisements on “general” KaDS and KaDS total scores was insignificant (*p* = 0.46 and *p* = 0.66, respectively).

## Discussion

Despite having minor overall benefits and emerging concerns about their safety, dietary supplements (DS) are consumed by roughly a half of the adult population of developed countries [2, 15, 35, 51, 52], with Poland not being any exception [53]. It is hypothesized that aggressive DS advertising shapes false beliefs among consumers to a certain extent and, consequently, drives supplement-taking behavior [14–16, 36]. The present study reports the development and validation of questionnaires to test knowledge about dietary supplements (KaDS) among the general public and to evaluate the trust in advertising them (TiADS). We also report a significant negative association between these constructs.

Knowledge about dietary supplements has been extensively studied in many population subgroups, including healthcare professionals [24, 54], medical students [52, 55], athletes [56] and the elderly [57]. However, few measures, if any, have been designed to assess commonly-identifiable beliefs, which are important from the perspective of public health, and few have used a rigorous methodology in the process of their development. The KaDS questionnaire reported in the current paper meets these needs. It was found to satisfactorily reflect the conceptual framework of the *a priori* defined construct and was refined to measure the possible aspects of knowledge commonly identified by the public. These advantages, together with its concise form (17 true/false items), make the tool suitable for screening the general population. Striving to preserve content validity of the questionnaire and keeping its limited length, however, was found to be in conflict with improving its psychometric properties, which is a common problem of short scales [58]. Indeed, the confirmatory factor analysis model fit and noncentrality-based indices as well as reliability estimates were modest and the loadings of some items low and even insignificant. Moreover, exploratory factor analysis with two factors could explain less than a third of variance. This all indicates that the nature of KaDS can hardly be limited to simply “general” and “specific”, nevertheless, adjusting the data to a more complex model was unjustified, as it could lead to misleading conclusions [59, 60]. Consequently, the presented 17-item KaDS test is likely inadequate for detailed knowledge evaluation and decision-making in individual respondents [58]. On the other hand, it appears suitable for rapid screening for research purposes. Indeed, similar short tests have already been used in research [61, 62].

Our findings indicate a strikingly low level of KaDS in the study group: the mean number of correctly assessed statements was close to a random guess. This raises substantial concerns regarding public health and highlights the need for adequate and evidence-based education about DS at all levels [57, 63, 64]. Even so, our findings remain in line with other similar reports, which conclude “knowledge (…) at a low level” [16], “deficits in consumer knowledge” about DS [65], “some knowledge deficiency” [52], “inadequate” and “limited” knowledge [55], “lack of knowledge or misinformation” [56], “substantial misconceptions” [57], and the presence of beliefs related to DS that “are rarely based on medical evidence” [15]. Surprisingly, the reported poor knowledge contrasts with the overestimated competence of the respondents, measured as self-reported familiarity with the statements. Such discrepancy may represent a cognitive bias identified by Kruger and Dunning [66], who argue that “people tend to hold overly favorable views of their abilities”. Intriguingly, this may be also attributed to the TiADS, which may lead to consolidation of illusory belief in having KaDS.

Trust is a multidimensional construct [34, 67], which presents a great challenge for its operationalization and quantification. A systematic and comprehensive analysis of “trust in advertising” was accomplished by Soh *et al*. [17]. The “trust in advertising dietary supplements” (TiADS) may have been unique and may require further refinement, as it applies to products directly related to human health–a value of great importance [68]. In addition, to reconcile several objectives of the present study, the measure should be concise and attainable. Similar existing measures employed by Menon *et al*. [69] or Huh *et al*. [70] for testing trust in online prescription drug information were not adopted for the present study due to their limited proof of validity. Instead, a new measure was developed from scratch. The TiADS questionnaire is an eight-item three-dimensional scale, which exhibits well-established content, criterion and concurrent validity as well as satisfactory internal consistency. The measure comprises “reliability” and “intelligibility” dimensions attributed to the cognitive domain and an “affect” factor, which reflects emotional domain. Contrary to similar measures [17], the present questionnaire does not include a behavioral domain; however, the items excluded from the measure, which theoretically reflected this domain, were found to correlate closely with the total TiADS questionnaire score. Furthermore, applying self-reported testing of claimed behavior may lead to biased results [71] and should be rather replaced with a more valid procedure employing tracking methods in consumer behavior research [72]. Apart from this, the item content of the TiADS questionnaire turned out to be largely similar to that of other measures [17, 70]. Nevertheless, it was noted that measuring trust in online prescription drug information based on different methods yields similar results [70].

The current study found overall trust in DS advertising to be low among the general public. This is intriguing in the light of high popularity of these products, but consistent with other research on DS advertising [5] and the reports on trust in online prescription drug information [69, 70]. Low trust in DS advertising also reflects the current global trend of growing crisis of trust [5]. Similarly to the above cited papers [69, 70], the TiADS in the current report was relatively stable across sociodemographic variables. The extent of trust in DS advertising was proportional to the frequency of DS use. In contrast, Huh *et al*. [70] report no relationship between trust in online prescription drug information and drug use. This may be explained by the fact that in contrast to DS, drugs are largely prescribed by doctors and not voluntarily selected by the consumer.

In 2017 Polish Supreme Audit Office (*orig*. Najwyższa Izba Kontroli) published an official report entitled *Marketing of dietary supplements* (*orig*. “Dopuszczanie do obrotu suplementów diety”), which highlighted numerous inefficiencies in the Polish national system of marketing, regulating and testing DS [16]. It proposes that inadequate monitoring of advertising plays a key role, with some advertisements offering unverifiable claims implying that DS may play a therapeutic role, and blurring the difference between the DS and over-the-counter medication. Another study performed in Poland found that 23 out of 27 advertisements recorded from TV or radio promised a beneficial effect, indicating benefits for organs or improved concentrations of biochemical indicators [36]. Such practices are against the law, which allows only certain and balanced health claims for DS advertising [73]. These findings in Poland are highly consistent with those obtained from other countries linking importunate marketing of DS with potential harm to the consumer [15, 35, 74, 75].

Our results confirm that extensive DS advertising is in conflict with the promotion of evidence-based knowledge about DS. A corresponding analogy to medical advertising was noted [76–81]: although many advertisements convey some factual information about drugs, they outweigh their benefits over the risks, undervalue lifestyle changes and employ emotional appeals [76]. These observations indicate that drug advertising [77], and perhaps that of dietary supplements offers little social value [82, 83]. As a consequence, our study, together with other Polish reports [16, 36, 83], emphasizes an urgent need to introduce more strict regulation and control over DS advertising, and even to consider a total ban on advertising them [83].

The current paper highlights a significant negative link between the level of KaDS and TiADS. This link was especially pronounced in people reporting not to take DS. The decision not to use DS may be a result of knowing much about them as well as holding little trust in their advertising. Other studies also suggest that the experience of consumers with dietary supplements may be a factor influencing reactions to new information about the product [84].

In contrast with TiADS, a negligible effect size was observed for the link between KaDS and having recent contact with DS advertisements, although this was statistically significant for “specific” knowledge. “Having contact” with DS advertising may only involve a fleeting interaction between a consumer and an advertisement. Consequently, such a contact may not be sufficient to substantially affect the knowledge about the advertised products, and a deeper commitment with the advert needs to be formed before trust can be built in the message [17, 85] thus justifying the acceptance of the advertising message [86]. Nevertheless, our findings indicate that having contact with DS advertising may strengthen the negative link between KaDS and TiADS, thus enhancing our understanding of mutual relationship between these factors.

Although an association was found between the KaDS and TiADS, this does not imply any causation, as highlighted by the Bradford Hill criteria [87]. In addition, a question on direction of potential causation remains also justified in this case [88]. The study design is incapable of providing a reply. Nevertheless, it is possible to speculate on the nature of the relationship between KaDS and TiADS:

1. Possessing prior KaDS (e.g. learned from healthcare specialists or acquired from professional literature or during biomedical courses) develops prejudice and distrust towards deceptive or ambiguous content of DS advertisement. A knowledgeable person seems to have no need to seek information about DS through advertising and may more accurately and critically assess its claims as untruthful or misleading [35, 89]. Trust is thought to develop from knowledge of the object of trust, and previous experiences with it [84, 90]. In fact, our present results suggest that medically educated people were found to trust in DS advertising significantly less than non-medically educated people, and one may consider a formal medical education as antecedent to trust in advertising health-related products.
2. This is also likely that the opposite relationship exists: the extent of TiADS shapes KaDS. It was suggested that individuals are more willing to process information from a trusted source [90]. This processing takes the form of attentiveness to facilitate learning [91, 92], increases involvement in advertising and decreases avoidance [17, 93]. This may allow trust in DS advertising to build and consolidate the illusory knowledge about the product. Such possibility is of great relevance for public health as it entails the need for continuous monitoring and strict control over DS advertising to foster evidence-based beliefs and encourage prudent behavior.
3. Above all, the link between the above discussed constructs may be more complex. Knowledge and trust may constitute joint effects of a common cause [88] such as sources of KaDS affecting attitudes to TiADS, or self-confidence playing a role in the consumer decision-making process. They may also mutually affect each other in a feedback loop and be mediated by multiple other factors.

Finally, our present findings may seem obvious. Yet the objectives of advertising, by definition, is the “presentation and promotion” of the product [31]. As DS are largely ineffective in health improvement, the only way to promote them appears to be by introducing manipulative messages about their utility, without facilitating informed health care decisions [94]. Thus, trusting in such messages is in opposition to true knowledge about them. This reasoning is simplified and one-sided, but underlines the deceptive nature of the content of DS advertisements and raises major concerns about ethical standards in their marketing [94, 95]. Moreover, the effect size of the KaDS-TiADS link is relatively high, appearing to be at least as high as the link between the presence, or absence of a formal medical education and KaDS. Eventually, in the light of a recent publication by Byron Sharp [96], which challenged common marketing theories, any hypothesis in marketing science is worth investigating. The present study offers new findings linking KaDS with TiADS, which may be of value to scientists, healthcare specialists, marketing experts and public health legislators.

The present study does have some limitations. Firstly, the samples used were drawn by non-probability methods, which may compromise the generalizability of the results. Secondly, most of the results are based on self-reported declarative data, which may further introduce substantial bias. Thirdly, the KaDS was compared with the TiADS in the same dataset as the final choice of the items to the KaDS test and no further validation of the knowledge test was performed. However, this questionnaire was constructed with the researchers being blinded to the result of TiADS questionnaire. Even so, the trust in advertising DS was still significantly negatively correlated with the sum of all 32 KaDS items considered for the final choice of the statements to the questionnaire. Finally, the developed questionnaires follow classical test theory, and do not take into account the difficulty of the test items expressed in the item response theory.

The current preliminary report requires further exploration confirming the results in a larger and randomly drawn dataset, seeking the mechanisms linking the observed constructs, and, ideally, testing the strategy to improve public knowledge about dietary supplements. It is advised to incorporate a mixture of qualitative and quantitative research methods, to gain a more comprehensive understanding of the reported phenomena.

## Conclusions

The developed questionnaires possess satisfactory validity when assessing knowledge about dietary supplements in the general public and their trust in advertising dietary supplements: both were found to be low in the study groups. The questionnaires appear to be adequate for screening purposes in research. A significant negative association was found between the two examined constructs, suggesting that extensive advertising of dietary supplements conflicts with the promotion of established facts regarding their properties. These results raise concerns regarding the level of knowledge of dietary supplements held by the general population as well as ethical standards in their marketing; they also suggest the need for educational campaigns regarding dietary supplements and their advertising, as well as for updated regulations, continuous monitoring and strict control over such advertising in order to build evidence-based beliefs and encourage responsible use. Our findings are only preliminary and require further confirmation and exploration to identify the mechanism linking the constructs and testing strategies to improve the existing situation.

## Supporting information

S1 Supporting Information Details on methods used in the development and validation of the questionnaire on knowledge about dietary supplements (KaDS). (DOC)

S2 Supporting Information Details on methods used in the development and validation of the questionnaire on trust in advertising dietary supplements (TiADS). (DOC)

S1 Survey Final survey “Dietary supplements and their advertising” subjected to 220 non-medically educated respondents. (DOCX)
