# Supplementary material for: Knowledge about dietary supplements and trust in advertising them: Development and validation of the questionnaires and preliminary results of the association between the constructs
Source: PLoS One. 2019 Jun 24;14(6):e0218398. doi: 10.1371/journal.pone.0218398 (PMC6590799; doi:10.1371/journal.pone.0218398)
Supplement: S1 Supporting Information — (DOC) [file pone.0218398.s001.DOC]

**S1 Supporting Information**

**Details on methods used in the development and validation of the questionnaire on knowledge about dietary supplements (KaDS)**

1. **Conceptual framework**
   1. “General” knowledge – defined as “familiarity with the useful facts concerning legal status of dietary supplements (DS) in general”.

Following concepts were highlighted:

- - 1. definition
    2. registration requirements
  1. “Specific” knowledge – defined as “familiarity with common, scientifically proven and useful facts about popular DS”.

Following concepts were highlighted:

- - 1. efficacy (special attention to this concept)
    2. safety
    3. pharmacokinetics
    4. interactions with medicines

1. **Development**
   1. Pre-development
      1. Interview 1

To gather only “common” and “popular” facts about DS, nine pharmacists and 17 people with no medical education were interviewed (22 women and four men, mean age ± standard deviation 40.4 ± 17.2 years, respondents of diverse education level, both users and non-users of DS) in a semi-structured way to establish the most commonly-purchased classes of DS, the ingredients which are the most identifiable by the public, and to collect common beliefs about DS in general.

- The pharmacists working in community pharmacies were asked: “what kind of DS people most frequently ask you for?”, “what kind of DS people most frequently purchase?”
- The laymen were asked: “What is a DS?”, “What kind of DS do you know?”, “What kind of DS do you use, if any? What for?”

As a result 11 ingredients of dietary supplements were found most identifiable:

- - - - vitamin C
      - magnesium (the first two were the most recognized DS)
      - vitamin D
      - calcium
      - iron
      - antioxidants
      - omega-3 fatty acids
      - probiotics
      - vitamin B_12_
      - glucosamine
      - fiber
    1. Interview 2

Open-ended questions were addressed to another group of 20 people with no medical education (12 women and eight men, age 33.5 ± 13.5 years, respondents of diverse education level, both users and non-users of DS) to identify public perceptions regarding the most common uses and risks related to the 11 identified earlier ingredients of DS. As a result, the interview identified the following most recognized uses of DS:

- - - - calcium for bone health (19 out of 20 responses)
      - vitamin C for immunity enhancement or cold prevention (18 out of 20 responses)
      - magnesium for muscle cramps (14 out of 20 responses)
      - vitamin D for bone health (14 out of 20 responses)
      - antioxidants for cancer prevention or free radical neutralization (11 out of 19 responses)
      - omega-3 fatty acids for cardiovascular disorders (eight out of 20 responses)

Risks related to the use of DS were much less recognizable with only three out of 20 responses linking vitamin C with renal disorders in vulnerable subjects.

- 1. Questionnaire formatting
     1. question stem – declarative sentence
     2. response format – binary (true/false). It was suggested that the use of smaller numbers of options (even with more items) results in more effortless testing and efficient use of testing time (Swanson *et al.* 2005), which was one of our priorities.
  2. Item generation

100 statements were drawn up following a rigorous review of legal documents as well as medical and scientific literature. The content of the statements was suited to the identified conceptual framework and was largely inspired by the results of pre-developmental interviews. The “scientifically proven” facts were established based on the results of at least two well-designed randomised controlled trials or, preferably, at least one systematic review with meta-analysis of such trials.

20 “general” and 80 “specific” statements were generated. They were intentionally developed as true (53 statements) or false (47 statements). Only the facts the panel believed were critically relevant for community health, i.e. “useful”, were gathered. The statements were reviewed by a specialist in Polish linguistics and further modified according to her suggestions.

- 1. Item reduction

A group of nine independent competent judges (medical doctors, pharmacists, and dieticians of both academic and clinical practice) were asked to review the statements. The judges were acquainted with the conceptual framework of the KaDS, but were blinded to the fact that KaDS is to be tested for association with the trust in advertising DS. The judges were asked to evaluate each statement in terms of its relevance and its value to public knowledge. A 5-point Likert scale was used for this purpose: from “5” (very relevant and important for the public to know) to “1” (very irrelevant and unimportant). A mean score was then generated for each statement. 59 statements marked by the judges less than “4” in average (three out of 20 “general” and 56 of 80 “specific” statements) were deleted, whereas the rest 41 were classified to further steps of questionnaire development process. The mean score awarded by the judges for each item retained in the final questionnaire is reported in Table 2 in the main text of the paper.

- 1. Item understanding

The retained 41 statements were tested with regard to their degree of understanding by the public. Four people with no medical education (two women and two men, age 43.5 ± 16.1 years, respondents of diverse education level) were asked (Hilton 2017):

- - 1. to speak in their own words how they understood each statement,
    2. to explain more difficult expressions (such as “antioxidant”, “probiotic, “left-handed”, “absorption”) in their own words,
    3. to respond whether the statement is true or false, to indicate how does a respondent arrive at this answer, and was this easy or difficult to answer.

The vast majority of the statements were understood as assumed by the panel of experts and were attainable to the respondents, but four required further clarification and were modified accordingly due to minor lexical problems listed in table below.

**Table. Modifications of the items resulting from the step of “item understanding"**

| No | Original statement | Modified statement | Comment |
| --- | --- | --- | --- |
| 1 | Vitamin and mineral supplements prevent diseases in healthy people. | Taking vitamin and mineral supplements prevents diseases in healthy people. | “Taking” is a prerequisite for any alleged action. |
| 2* | Supplementation with high doses of calcium diminishes the absorption of magnesium from the food. | The use of high doses of calcium diminishes the absorption of magnesium from the food. | The expression “supplementation with” was not fully clear to some respondents and was simplified accordingly. |
| 3 | In the elderly, supplementation with magnesium preparations prevents muscle cramps | In the elderly, the use of magnesium preparations prevents muscle cramps | See above |
| 4 | Regular use of vitamin C reduces the risk of catching a cold. | Regular use of vitamin C reduces the risk of catching a cold. | The expression “zachorować na przeziębienie” was simplified to “przeziębienie”, which sounds more natural in Polish without losing its meaning. |

* a statement rejected during the subsequent questionnaire design process

1. **Pre-testing**

As the statements in the KaDS questionnaire were assumed to be “popular”, reflecting “common” knowledge, the process of questionnaire pre-testing aimed at identifying the statements, which are not familiar to the general public. The retained 41 randomly-ordered statements were preceded with a suitable cover letter highlighting the rationale for the survey. Such survey was subjected to 57 people with no medical education (45 women and 12 men, age 37.9 ± 11.9 years, respondents of diverse education level, both users and non-users of DS) through a web-based test based on Google Forms (Google, Mountain View, CA, USA). The respondents were asked to assess whether each statement was “true”, “false” or “don’t know”. Statements with more than 50% of “don’t know” answers were stated as “unfamiliar to the public”, and were excluded from further analyses.

- - - - Respondent recruitment – judgmental and snowball (non-probability) sampling technique: diverse relatives and friends of the researchers with no medical education and no link to academia were recruited and asked to further snowball the questionnaire.
      - Response rate – was not determined in any of the web-based questionnaires across the study due to the incalculable number of invited people.

Four out of the 41 tested statements were assessed as unfamiliar to the public and excluded from further analyses. These four unfamiliar statements (all “specific”) comprised mainly the aspects of “interactions with medicines” and “pharmacokinetics”:

- - - - “Taking magnesium can limit the absorption of oral antibiotics” (true) with 43 out of 56 (77%) “don’t know” responses
      - “Zinc, calcium, magnesium and iron supplements limit the absorption of some antibiotics” (true) with 42 out of 56 (75%) “don’t know” responses
      - “The use of high doses of calcium diminishes the absorption of magnesium from the food” (true) with 34 out of 57 (60%) “don’t know” responses
      - “Left-handed vitamin C is the only biologically active form of vitamin C” (false) with 30 out of 57 (53%) “don’t know” responses.

Familiarity with each item retained in the final questionnaire is presented in Table 2 in the main text of the paper.

1. **Testing**

A web-based form (Google Form) of the draft of the KaDS questionnaire with 37 retained items and with true/false response format (no “don’t know” option any more) was subjected to a sample of 220 people with no medical education (characteristics of this sample of respondents was reported in Table 1 in the main text of the paper). The recruitment strategy was the same as described above and the response rate was not determined for the same reason as above (see III. Pre-testing).

1. Keeping conceptual framework of the scale in mind in order to assure content validity, questionnaire purification and refinement was performed basing on the following psychometric parameters:
   - 1. item difficulty – excessively difficult (> 90% of wrong answers) and easy (< 10% of wrong answers) statements were excluded.

The results of testing stage showed that four statements (all “general”) were deemed too easy to be included to the final questionnaire:

- - - - “Dietary supplements can completely compensate for the deficiencies resulting from a poor or defective diet” (false) with 13 out of 219 (5.9%) wrong answers
      - “Taking too many dietary supplements can be harmful to your health” (true) with 17 out of 219 (7.8%) wrong answers
      - “Taking supplements may replace the need for a varied and balanced diet” (false) with 21 wrong answers out of 219 (9.6%)
      - “You don’t need to inform your doctor about taken dietary supplements” (false) with 21 wrong answers out of 219 (9.6%).

Additionally, one “specific” statement was deemed too difficult to be included to the final questionnaire:

- “Taking omega-3 fatty acids preparations protects against heart disease” (false) with 199 wrong answers out of 220 (90.5%).

Thus, this step generated a set of 32 statements: 13 “general” and 19 “specific”. The difficulty of each item retained in the final questionnaire is reported in Table 2 in the main text of the paper.

- - 1. item discrimination – statements with an item discrimination (measured as a corrected item-total correlation with assumed subscales) of less than 0.20 were excluded.
- Mean correlation coefficient of all 13 “general” items was 0.19 (range: -0.04 to 0.52). With the use of backward stepwise procedure, the statements with the lowest item discrimination values was excluded to yield seven substantially correlated ones.
- 19 “specific” statements were much less correlated with each other than the “general” statements (mean correlation coefficient: -0.01, range: -0.38 to 0.21). Similarly, the backward stepwise elimination allowed for selecting seven substantially correlated ones.
  - 1. maximization of internal consistency

The goal to maximize the internal consistencies of the subscales was convergent with the goal to select the statements with highest item discrimination. No action was performed at this step.

- - 1. balancing the number of “true” and “false” correct answers with content reanalysis

This was suggested that people completing a multiple choice test and finding a few subsequent correct answers located in the same position are likely to expect the next correct answer in the other position. Consequently, the positions of correct answers may be intentionally balanced by the authors of the test to overcome the possible bias related to correct answer location (Bar-Hillel and Attali 2002).

- The number of “true” and “false” correct answers in the seven retained “general” statements was relatively similar (two “true and five “false”) and they logically reflected the *a priori* set conceptual framework of the scale, assuring its content validity.
- The correct answer for all the seven retained “specific” items was “false”. Because of that uniformity, content reanalysis of retained statements was performed to show that they all reflected only the “efficacy” concept of “specific” KaDS. As a result, three more items were reincluded to the “specific” scale to preserve its versatility and restore content validity: two representing the “safety” domain and one representing the “pharmacokinetics” domain. The correct answer for two of the reincluded statements was “true”. Such a decision to expand the “specific” subscale, basing mainly on the content analysis, compromised psychometric parameters of the scale, but was justified by the need to keep an *a priori* set conceptual framework. Finally, the “specific” subscale of the KaDS was set as 10-item.

1. To ensure the criterion validity of the finally designed 17-item questionnaire, the KaDS test was also applied to 121 medically educated people: 77 medical students (59 women and 18 men, age 22.8 ± 2.0 years) and 44 healthcare practitioners (35 women and nine men, age 34.8 ± 9.6 years).
   - - - Respondent recruitment – convenience (non-probability) sampling: the invitation to participate in the web-based test was put on the social media (Facebook, Menlo Park, CA, USA) of Department of Pharmacology and Toxicology, Medical University of Lodz.
       - Response rate – not determined for the same reason as above (see III. Pre-testing).

The KaDS test was also combined with the measure of “trust in advertising dietary supplements” to assess the association between the KaDS and trust in advertising DS.

The criterion validity of the KaDS questionnaire was checked in two ways:

- comparing the mean test score between medically and non-medically educated people
- assessing the correlation between the KaDS test score and a year of study among a group of medical students.

The psychometric characteristics of the final KaDS questionnaire, including internal consistencies of the subscales and the results of exploratory and confirmatory factor analysis, is presented in Table 2 in the main text of the paper.

**References**

Bar-Hillel M, Attali Y. Seek whence: Answer sequences and their consequences in key-balanced multiple-choice tests. The American Statistician. 2002; 56(4): 299-303

Hilton CE. The importance of pretesting questionnaires: a field research example of cognitive pretesting the Exercise referral Quality of Life Scale (ER-QLS). Int J Soc Res Methodol. 2017; 20(1): 21-34.

Swanson DB, Holtzman KZ, Clauser BE, Sawhill AJ. Psychometric characteristics and response times for one-best-answer questions in relation to number and source of options. Acad Med. 2005; 80(10 Suppl): S93-6.
