# Supplementary material for: Knowledge about dietary supplements and trust in advertising them: Development and validation of the questionnaires and preliminary results of the association between the constructs
Source: PLoS One. 2019 Jun 24;14(6):e0218398. doi: 10.1371/journal.pone.0218398 (PMC6590799; doi:10.1371/journal.pone.0218398)
Supplement: S2 Supporting Information — (DOC) [file pone.0218398.s002.DOC]

**S2 Supporting Information**

**Details on methods used in the development and validation of the questionnaire on trust in advertising dietary supplements (TiADS)**

1. **Conceptual framework**

TiADS – defined as “a belief that advertising is a credible source of information about dietary supplements, arouses emotions and willingness to act on the basis of information conveyed by advertisements”.

The definition provided by Soh *et al.* (2009) was adopted; however, the word “confidence” was replaced with “belief” in order to emphasize the cognitive character of trust.

The following domains were highlighted:

- cognitive
- behavioral
- emotional

1. **Development**
   1. Pre-development
   2. Questionnaire formatting

A semantic differential scale was chosen. In measuring positive psychological constructs such as trust, semantic differential format may provide more consistent results than Likert scale. Semantic differential format may effectively reduce acquiescence bias without lowering psychometric quality (Friborg *et al.* 2006).

- 1. Item generation

The 20 developed items largely belonged to the evaluative factor with regard to Osgood *et al.* (1957), *i.e.* good-bad, pleasant-unpleasant, or positive-negative. Their content is presented in the table below.

**Table. Item review in the development of the trust in advertising dietary supplements questionnaire.** The items presented on gray background were retained in the final eight-item questionnaire.

| Domain | Number | Expressions generated | Non-professional item review | Professional item review | Pre-testing |
| --- | --- | --- | --- | --- | --- |
| Cognitive | 1 | true - untrue |  | merged |  |
|  | 2 | reliable - unreliable |  |  |  |
|  | 3 | credible - not trustworthy |  |  |  |
|  | 4 | full - incomplete |  | merged |  |
|  | 5 | comprehensive - incomprehensive |  |  |  |
|  | 6 | understandable - unclear |  |  |  |
|  | 7 | unambiguous - ambiguous |  |  |  |
|  | 8 | trustworthy - deceptive |  | excluded |  |
|  | 9 | transparent - tangled |  | excluded |  |
|  | 10 | truthful - deceitful | excluded |  |  |
|  | 11 | authentic - inauthentic |  | excluded |  |
|  | 12 | rational - irrational |  | excluded |  |
|  |  | convincing - unconvincing | included | excluded |  |
| Behavioral | 1 | they’re useful - they’re unnecessary |  |  | excluded |
|  | 2 | they help me decide to buy - they make it difficult to make a purchase decision |  |  | excluded |
|  | 3 | they help me take care of my health - they make it difficult to take care of my health |  |  | excluded |
|  | 4 | you cannot live without them - they are needless | excluded |  |  |
| Emotional | 1 | I like them - I don’t like them |  |  |  |
|  | 2 | they're cool - they're awful |  | excluded |  |
|  | 3 | I enjoy them - they annoy me |  |  |  |
|  | 4 | they should be broadcast more often - their emission should be limited |  |  |  |
| The number of items retained at each step | | 20 | 19 | 11 | 8 |

- 1. Non-professional item review and understanding

Five non-specialists (four women and one man, age 38.8 ± 14.9 years, respondents of diverse education level, both users and non-users of DS) were recruited to review the developed items. In their opinion, the majority of the items were satisfactorily understandable and relevant to DS advertising. Two of the items were found irrelevant/redundant and excluded from further processing (see table above). One additional item was proposed by non-specialists, which was initially accepted by the panel of experts (see table above) and this was included to further analyses.

- 1. Professional item review

The assessment of the questionnaire by the competent judges (psychologists and marketing specialists) led to a reduction of the number of items to 11. In the cognitive domain of the questionnaire, the judges suggested merging two pairs of items due to their semantic proximity (see table above). Additionally, five other cognitive items were deleted, as they were graded as less relevant than the others. This resulted in five items being retained in the cognitive domain. Similarly, one item from the emotional domain was deleted, resulting in three items being preserved in emotional domain. None of the three items belonging to the behavioral domain was deleted.

1. **Pre-testing**
   1. Structure assessment and forming the final questionnaire

11-item draft of TiADS questionnaire was administered to 174 respondents in a web-based form for pre-testing and item purification and refinement. The respondents (121 women and 53 men, age 30.0 ± 10.5 years) included 77 non-medically educated people, 51 medical students and 46 healthcare practitioners.

- - - - Respondent recruitment – convenience, judgmental and snowball (non-probability) sampling technique: respondent were recruited through the social media (Facebook, Menlo Park, CA, USA) of Department of Pharmacology and Toxicology, Medical University of Lodz as well as through diverse relatives and friends of the researchers with further request to snowball the survey.
      - Response rate – was not determined in any of the web-based questionnaires across the study due to the incalculable number of invited people.

The choice of the items to be retained in the final TiADS questionnaire was based on the results of exploratory factor analysis (EFA) with subsequent internal consistency assessment. Varimax rotation was used to enhance the dissimilarity of the extracted factors in EFA. The number of factors were determined based on the scree-plot and Kaiser's criterion (eigenvalue above 1). Redundant items were eliminated in a stepwise manner to maximize the primary factor loadings (> 0.5) of the retained items and minimize their cross-loadings (< 0.3). Internal consistency was estimated with Cronbach’s alpha and McDonald’s omega.

EFA resulted in a four-factor solution explaining 75.6% of the total variance. Three inconsistent items were stepwise removed (see table above) to purify a measure to three-factor structure of eight items explaining 78.6% of the variance (for the results of EFA see Table 3 in the main text of the paper). All the removed items belonged to the behavioral domain of the construct. Although they were removed, their sum strongly correlated with the sum of the retained items (r=0.59, 95%CI: 0.48-0.68, *p*<0.0001). The final list of factors demonstrated satisfactory internal consistency and were named “reliability” (three items of Cronbach’s alpha 0.85), “intelligibility” (two items of Cronbach’s alpha 0.60), and “affect” (three items of Cronbach’s alpha 0.88). The final eight-item TiADS questionnaire exhibited acceptable distribution of the responses: skewness 0.43 (95%CI: 0.07 to 0.79) and kurtosis -0.16 (95%CI: -0.87 to 0.56).

- 1. Criterion validity

To assess the criterion validity of the TiADS questionnaire, the TiADS scores was compared with the results of Polish-version ADTRUST measure, which was added to the survey subjected to 174 respondents in the pre-testing stage of the questionnaire development.

The ADTRUST is a measure of “trust in advertising” developed by Soh *et al.* (2009). This is a 20-item scale with four distinct factors: reliability, usefulness, affect and willingness to rely on advertising. It exhibits high reliability and validity; however, it is generalized to the advertising of all goods, services and ideas. The Polish-version of ADTRUST was prepared for the purpose of the current study. The original tool was independently translated to Polish by two Polish people proficient in English: a pharmacist with academic experience and a psychologist. The two translations were compared, discussed and a final Polish-version was chosen. Following this, a back-translation was performed by a professional bilingual Polish-English translator. A back-translated version was not found to be substantially different than the original questionnaire. Three people recruited from the public (three women, age 39.3 ± 18.9 years, respondents of diverse education level) found the Polish-version ADTRUST to be understandable and unambiguous.

- 1. Concurrent validity

The answer to the question of “how much does a respondent trust the information conveyed in dietary supplement advertising” was recorded on a seven-point scale with anchors of ”absolutely trust” (7) - “don’t trust at all” (1). This question was also added to the survey subjected to 174 people in the pre-testing stage. It was used as a non-diagnostic single-item measure of “trust in advertising dietary supplements” to assess concurrent validity of the developed measure.

1. **Testing**

A web-based form (Google Form) of the final eight-item TiADS questionnaire (combined with the 37-item draft version of the test on knowledge about dietary supplements) was subjected to a sample of 220 people with no medical education (characteristics of this sample of respondents was reported in Table 1 in the main text of the paper) and additionally to 121 medically-educated people. Recruitment strategy was the same as described above and the response rate was not determined for the same reason as above (see III. Pre-testing). Confirmatory factor analysis was used to verify the established before structure of the final eight-item TiADS questionnaire. Internal consistency was again estimated (Cronbach’s alphas and McDonald’s omega) as well as distribution of the responses (for results see Table 3 in the main text of the paper).

**References**

Friborg O, Martinussen M, Rosenvinge JH. Likert-based vs. semantic differential-based scorings of positive psychological constructs: A psychometric comparison of two versions of a scale measuring resilience. Pers Individ Dif. 2006; 40(5): 873-84.

Osgood CE, Suci GJ, Tannenbaum PH. The measurement of meaning. Oxford, England: Univer. Illinois Press; 1957.

Soh H, Reid LN, King KW. Measuring Trust In Advertising. J Advert. 2009; 38(2): 83-104.
