# Supplementary material for: Knowledge about dietary supplements and trust in advertising them: Development and validation of the questionnaires and preliminary results of the association between the constructs
Source: PLoS One. 2019 Jun 24;14(6):e0218398. doi: 10.1371/journal.pone.0218398 (PMC6590799; doi:10.1371/journal.pone.0218398)
Supplement: S1 Survey — (DOCX) [file pone.0218398.s003.docx]

**S1 Survey**

Dietary supplements and their advertising

1. Knowledge about dietary supplements

Below, there are 37 statements^[[1]](#footnote-1)^ about dietary supplements. Please indicate whether the statement is true of false. Please give your answer according to your beliefs - do not look for answers on the Internet or in textbooks. Just mark it as you think - even if you're not 100% sure.

1. **Taking vitamin and mineral supplements prevents diseases in healthy people.**

- True
- False

1. All forms of magnesium are equally well absorbed.

- True
- False

1. Dietary supplements can completely compensate for the deficiencies resulting from a poor or defective diet.

- True
- False

1. **In the elderly, the use of magnesium preparations prevents muscle cramps.**

- True
- False

1. Ingesting vitamin D with fatty foods improves its absorption.

- True
- False

1. The use of cranberry preparations by women prevents urinary tract infections.

- True
- False

1. **In the elderly, taking vitamin D reduces the risk of bone fractures.**

- True
- False

1. Dietary supplements may interfere with the effects of medicines prescribed by a doctor.

- True
- False

1. Taking probiotics reduces the duration of antibiotic-associated diarrhea.

- True
- False

1. **Before being marketed, dietary supplements must be tested for efficacy and safety.**

- True
- False

1. **Taking dietary supplements containing calcium reduces the risk of bone fractures in the elderly.**

- True
- False

1. **An ingredient may be sold both as a medicine or as a dietary supplement.**

- True
- False

1. **The quality of dietary supplements is routinely tested before being marketed.**

- True
- False

1. **The packaging of dietary supplements must contain information on possible adverse effects resulting from their use.**

- True
- False

1. **Taking excessive amounts of magnesium supplements can cause diarrhea and nausea.**

- True
- False

1. **The use of multivitamin preparations protects against heart diseases.**

- True
- False

1. **Vitamin C naturally present in food is better assimilated than synthetic.**

- True
- False

1. Dietary supplements are intended only for adults.

- True
- False

1. The purpose for using dietary supplements is to prevent some diseases.

- True
- False

1. The use of iron preparations treats anemia.

- True
- False

1. The use of vitamin C prevents the development of cancer.

- True
- False

1. You don’t need to inform your doctor about taken dietary supplements.

- True
- False

1. Supplements containing vitamin D should not be taken during the summer months because of the risk of overdose.

- True
- False

1. The use of dietary supplements may interfere with the treatment of chronic diseases (e.g. hypertension, diabetes).

- True
- False

1. **Dietary supplements are food.**

- True
- False

1. **The use of antioxidants prevents the development of cancer.**

- True
- False

1. Dietary supplements may contain substances not found naturally in food.

- True
- False

1. **Regular use of vitamin C reduces the risk of catching a cold.**

- True
- False

1. Taking omega-3 fatty acids preparations protects against heart disease.

- True
- False

1. **Dietary supplement registration requires assessing the composition of the product by the appropriate supervisory body.**

- True
- False

1. **People with kidney disease should not use high doses of vitamin C.**

- True
- False

1. Dietary supplements are a type of medication used to treat mild diseases.

- True
- False

1. Taking dietary supplements containing fiber by obese persons reduces body weight.

- True
- False

1. People regularly taking vitamin C suffer from less severe colds.

- True
- False

1. **All dietary supplements sold in pharmacies have been tested for safety.**

- True
- False

1. Taking too many dietary supplements can be harmful to your health.

- True
- False

1. Taking supplements may replace the need for a varied and balanced diet.

- True
- False

1. Advertising of dietary supplements
2. You can find advertisements of dietary supplements on television, radio, the Internet, the press and many other media. During the last week, did you encounter advertisements of dietary supplements?

- No
- Yes

1. We would like to know your opinion about the information conveyed by the advertisements of dietary supplements. Considering the advertisements you have encountered in the last six months, select the score that best describes your answer.

Each scale consists of 5 points, where:

1 means "I strongly agree with the term on the leftmost side of the scale",

2 means "I agree with the term on the leftmost side of the scale",

3 means "neither agree nor disagree",

4 means "I agree with the term on the rightmost side of the scale",

5 means "I strongly agree with the term on the far right side of the scale".

1. The information conveyed by the advertisements of dietary supplements are:

True (reliable) – 1 – 2 – 3 – 4 – 5 – Untrue (unreliable)

Credible – 1 – 2 – 3 – 4 – 5 – Not trustworthy

Full (comprehensive) – 1 – 2 – 3 – 4 – 5 – Incomplete (incomprehensive)

Understandable – 1 – 2 – 3 – 4 – 5 – Unclear

Unambiguous – 1 – 2 – 3 – 4 – 5 – Ambiguous

1. Advertisements of dietary supplements:

I like them – 1 – 2 – 3 – 4 – 5 – I don’t like them

I enjoy them – 1 – 2 – 3 – 4 – 5 – They annoy me

They should be broadcast more often – 1 – 2 – 3 – 4 – 5 – Their emission should be limited

1. At the very end, please provide basic data about yourself:
2. Do you take dietary supplements?

- No
- Yes, from time to time
- Yes, everyday

1. Socioeconomic status (how do you subjectively describe your social and material status?)

- Low
- Middle
- High

1. Age [years]: __________________
2. Sex

- Male
- Female

1. Education

- Primary
- Secondary
- Higher – Bachelor
- Higher – Master
- Higher – Doctorate

Suplementy diety i ich reklama

1. Wiedza na temat suplementów diety

Ułożyliśmy 37 stwierdzeń^[[2]](#footnote-2)^ dotyczących suplementów diety. Prosimy Cię o wskazanie, czy dane stwierdzenie jest prawdziwe, czy też fałszywe. Prosimy o odpowiedź według Twoich przekonań - nie poszukuj odpowiedzi w Internecie czy mądrych książkach. Po prostu zaznacz tak, jak sądzisz - nawet jeśli nie jesteś tego stuprocentowo pewien.

1. **Przyjmowanie witaminowych i mineralnych suplementów diety zapobiega chorobom u zdrowych osób.**

- Prawda
- Fałsz

1. Wszystkie postacie magnezu wchłaniają się tak samo.

- Prawda
- Fałsz

1. Stosując suplementy diety, można całkowicie zrekompensować braki wynikające z ubogiej lub niepełnowartościowej diety.

- Prawda
- Fałsz

1. **U osób starszych stosowanie preparatów magnezu zapobiega skurczom mięśni.**

- Prawda
- Fałsz

1. Przyjmowanie witaminy D z tłustymi pokarmami poprawia jej wchłanianie.

- Prawda
- Fałsz

1. Stosowanie przez kobiety preparatów z żurawiną zapobiega infekcjom dróg moczowych.

- Prawda
- Fałsz

1. **U osób starszych przyjmowanie witaminy D zmniejsza ryzyko złamań kości.**

- Prawda
- Fałsz

1. Suplementy diety mogą zaburzać działanie leków przepisanych przez lekarza.

- Prawda
- Fałsz

1. Przyjmowanie probiotyków skraca czas trwania biegunki poantybiotykowej.

- Prawda
- Fałsz

1. **Przed wprowadzeniem na rynek suplementy diety muszą zostać zbadane pod kątem skuteczności i bezpieczeństwa.**

- Prawda
- Fałsz

1. **Przyjmowanie przez osoby starsze suplementów diety zawierających wapń zmniejsza u nich ryzyko złamań kości.**

- Prawda
- Fałsz

1. **Ta sama substancja może być sprzedawana zarówno w postaci leku, jak i suplementu diety.**

- Prawda
- Fałsz

1. **Jakość suplementów diety jest rutynowo badana przed dopuszczeniem ich do obrotu.**

- Prawda
- Fałsz

1. **Na opakowaniach suplementów diety musi znajdować się informacja na temat możliwych skutków ubocznych wynikających z ich stosowania.**

- Prawda
- Fałsz

1. **Przyjmowanie nadmiernej ilości suplementów diety zawierających magnez może spowodować biegunkę i nudności.**

- Prawda
- Fałsz

1. **Stosowanie preparatów multiwitaminowych chroni przed chorobami serca.**

- Prawda
- Fałsz

1. **Witamina C naturalnie występująca w pożywieniu jest lepiej przyswajalna niż syntetyczna.**

- Prawda
- Fałsz

1. Suplementy diety przeznaczone są tylko dla osób dorosłych.

- Prawda
- Fałsz

1. Celem stosowania suplementów diety jest zapobieganie niektórym chorobom.

- Prawda
- Fałsz

1. Stosowanie preparatów żelaza leczy anemię.

- Prawda
- Fałsz

1. Przyjmowanie witaminy C chroni przed zachorowaniem na nowotwór.

- Prawda
- Fałsz

1. O stosowanych suplementach diety nie trzeba wspominać lekarzowi.

- Prawda
- Fałsz

1. Suplementów zawierających witaminę D nie należy przyjmować w miesiącach letnich ze względu na ryzyko przedawkowania.

- Prawda
- Fałsz

1. Stosowanie suplementów diety może zaburzać leczenie chorób przewlekłych (np. nadciśnienia, cukrzycy).

- Prawda
- Fałsz

1. **Suplementy diety to środki spożywcze.**

- Prawda
- Fałsz

1. **Stosowanie antyoksydantów zapobiega rozwojowi nowotworów.**

- Prawda
- Fałsz

1. Suplementy diety mogą zawierać substancje niewystępujące naturalnie w żywności.

- Prawda
- Fałsz

1. **Regularne stosowanie witaminy C zmniejsza ryzyko przeziębienia.**

- Prawda
- Fałsz

1. Przyjmowanie preparatów omega-3 chroni przed chorobami serca.

- Prawda
- Fałsz

1. **Wprowadzenie suplementu diety do sprzedaży wymaga skontrolowania składu produktu przez odpowiedni organ nadzorczy.**

- Prawda
- Fałsz

1. **Osoby z chorobami nerek nie powinny stosować wysokich dawek witaminy C.**

- Prawda
- Fałsz

1. Suplementy diety to rodzaj leków służących do leczenia łagodnych schorzeń.

- Prawda
- Fałsz

1. Przyjmowanie przez osoby otyłe suplementów diety zawierających błonnik zmniejsza masę ciała.

- Prawda
- Fałsz

1. Osoby regularnie zażywające witaminę C przechodzą przeziębienia łagodniej.

- Prawda
- Fałsz

1. **Wszystkie suplementy diety sprzedawane w aptekach zostały przebadane pod kątem bezpieczeństwa.**

- Prawda
- Fałsz

1. Przyjmowanie zbyt wielu suplementów diety może wywierać szkodliwy wpływ na zdrowie.

- Prawda
- Fałsz

1. Stosowanie suplementów diety może być substytutem (zamiennikiem) zróżnicowanej diety.

- Prawda
- Fałsz

1. Reklama suplementów diety
2. Z reklamami suplementów diety możesz spotkać się w telewizji, radiu, Internecie, prasie i w wielu innych mediach. Czy w ostatnim tygodniu spotkałeś(aś) się z reklamą suplementów diety?

- Nie
- Tak

1. Chcielibyśmy teraz poznać Twoją opinię dotyczącą komunikatów zawartych w reklamach suplementów diety. Biorąc pod uwagę reklamy, z którymi miałeś(łaś) do czynienia w ciągu ostatnich sześciu miesięcy, zaznacz na skali określenie, które najlepiej odpowiada na pytanie.

Każda skala składa się z 5 punktów, gdzie:

1 oznacza „zdecydowanie zgadzam się z określeniem znajdującym się skrajnie z lewej strony skali”,

2 oznacza „zgadzam się z określeniem znajdującym się skrajnie z lewej strony skali”,

3 oznacza „ani się zgadzam, ani nie zgadzam”,

4 oznacza „zgadzam się z określeniem znajdującym się skrajnie z prawej strony skali”,

5 oznacza „zdecydowanie zgadzam się z określeniem znajdującym się skrajnie z prawej strony skali”.

1. Informacje zawarte w reklamach suplementów diety są:

Prawdziwe (rzetelne) – 1 – 2 – 3 – 4 – 5 – Nieprawdziwe (nierzetelne)

Wiarygodne – 1 – 2 – 3 – 4 – 5 – Niewiarygodne

Pełne (kompleksowe) – 1 – 2 – 3 – 4 – 5 – Niepełne (niekompleksowe)

Zrozumiałe – 1 – 2 – 3 – 4 – 5 – Niezrozumiałe

Jednoznaczne – 1 – 2 – 3 – 4 – 5 – Niejednoznaczne

1. Reklamy suplementów diety:

Lubię je – 1 – 2 – 3 – 4 – 5 – Nie lubię ich

Sprawiają mi przyjemność – 1 – 2 – 3 – 4 – 5 – Irytują mnie

Powinny być częściej emitowane – 1 – 2 – 3 – 4 – 5 – Powinno się ograniczyć ich emisję

1. Na sam koniec prosimy o podanie podstawowych danych o sobie::
2. Czy przyjmujesz suplementy diety?

- Nie
- Tak, od czasu do czasu
- Tak, codziennie

1. Status socjoekonomiczny (jak subiektywnie opiszesz swój status społeczny i materialny?)

- Niski
- Średni
- Wysoki

1. Wiek [w latach]: __________________
2. Płeć

- Mężczyzna
- Kobieta

1. Wykształcenie

- Podstawowe
- Średnie lub zawodowe
- Wyższe – licencjat
- Wyższe – magister
- Wyższe – doktor

1. The statements are presented in the order as they appear in the questionnaire. The statements in bold were retained in the final 17-item knowledge test. [↑](#footnote-ref-1)
2. The statements are presented in the order as they appear in the questionnaire. The statements in bold were retained in the final 17-item knowledge test. [↑](#footnote-ref-2)
